# Vector competence of *Culex quinquefasciatus* for Tembusu virus and viral factors for virus transmission by mosquitoes

**DOI:** 10.1186/s13567-024-01361-3

**Published:** 2024-09-18

**Authors:** Yibin Tang, Yu He, Xiaoli Wang, Zhen Wu, Senyan Du, Mingshu Wang, Renyong Jia, Dekang Zhu, Mafeng Liu, Xinxin Zhao, Qiao Yang, Ying Wu, Shaqiu Zhang, Juan Huang, Xumin Ou, Di Sun, Anchun Cheng, Shun Chen

**Affiliations:** 1 Institute of Veterinary Medicine and Immunology, Sichuan Agricultural University, Chengdu, 611130 Sichuan China; 2 Research Center of Avian Disease, College of Veterinary Medicine, Sichuan Agricultural University, Chengdu, 611130 Sichuan China; 3 Key Laboratory of Animal Disease and Human Health of Sichuan Province, Sichuan Agricultural University, Chengdu, 611130 Sichuan China; 4 Key Laboratory of Agricultural Bioinformatics, Ministry of Education, Chengdu, 611130 Sichuan China; 5 Research Center for Swine Diseases, Sichuan Agricultural University, Chengdu, 611130 Sichuan China

**Keywords:** Tembusu virus, *Culex quinquefasciatus*, vector competence, mosquito transmission, vertical transmission, venereal transmission

## Abstract

The ongoing epidemic of flaviviruses worldwide has underscored the importance of studying flavivirus vector competence, considering their close association with mosquito vectors. Tembusu virus is an avian-related mosquito-borne flavivirus that has been an epidemic in China and Southeast Asia since 2010. However, the reason for the outbreak of Tembusu virus in 2010 remains unclear, and it is unknown whether changes in vector transmission played an essential role in this process. To address these questions, we conducted a study using *Culex quinquefasciatus* as a model for Tembusu virus infection, employing both oral infection and microinjection methods. Our findings confirmed that both vertical and venereal transmission collectively contribute to the cycle of Tembusu virus within the mosquito population, with persistent infections observed. Importantly, our data revealed that the prototypical Tembusu virus MM_1775 strain exhibited significantly greater infectivity and transmission rates in mosquitoes than did the duck Tembusu virus (CQW1 strain). Furthermore, we revealed that the viral E protein and 3′ untranslated region are key elements responsible for these differences. In conclusion, our study sheds light on mosquito transmission of Tembusu virus and provides valuable insights into the factors influencing its infectivity and transmission rates. These findings contribute to a better understanding of Tembusu virus epidemiology and can potentially aid in the development of strategies to control its spread.

## Introduction

Mosquito-borne flaviviruses, such as Zika virus (ZIKV), Dengue virus (DENV), Japanese encephalitis virus (JEV), and yellow fever virus (YFV), pose significant threats to global public health, causing various diseases in animals and humans worldwide. Among these flaviviruses, Tembusu virus (TMUV) is an emerging mosquito-borne flavivirus that causes severe neurological and reproductive diseases in birds.

TMUV (MM_1775 strain) was first isolated from *Cx. tritaeniorhynchus* mosquitoes in Malaysia in 1955 [1]. However, it was only sporadically reported in subsequent decades. In 2010, an infectious disease characterized by duck egg-drop syndrome broke out in China, resulting in significant economic losses. The pathogen responsible for this outbreak was eventually confirmed as duck TMUV. Currently, TMUV strains are classified into three clusters based on their major antigen gene E [2, 3]. Cluster 1 includes TMUV strains isolated from Southeast Asia, while Cluster 2 consists of most waterfowl-origin isolates from China and Southeast Asian countries. Most of the duck TMUV isolated in China since 2010 fall into Cluster 2.2. The mosquito-origin TMUV, including the MM_1775 strain, and the recent chicken-origin TMUV (since 2020) form Cluster 3 [2].

TMUV exhibits broad host tropism and has been isolated from mosquitoes and various avian species, including ducks, chickens, geese, and sparrows. In laboratory studies, TMUV has shown efficient replication in avian (i.e., DF-1, embryo fibroblasts of duck and goose), mosquito (i.e., C6/36), and mammalian (i.e., Vero, BHK-21, HEK293, HepG2 and SH-SY5Y) cell lines. Although TMUV exhibits high neurovirulence in mice, it does not show neuroinvasiveness in adult mice. Serological investigations in China [4] and Thailand [5] have detected high anti-TMUV antibody titres in individuals at risk, such as workers on duck farms or nearby residents, and a high positive rate of TMUV by RT-PCR in duck farm workers in China has also been reported [4]. This raises the possibility that TMUV is a potentially zoonotic flavivirus.

Most mosquito-borne flaviviruses, such as ZIKV and DENV, undergo a cycle between mosquitoes and vertebrate hosts in nature. When mosquitoes bite and feed on infected hosts, they acquire virions that circulate in the host's blood. Then, the viruses establish an infection in the mosquito midgut and disseminate to other organs through the mosquito haemocoel. After robust virus replication in the salivary glands, the virus can be transmitted to naive hosts through mosquito biting. Within the mosquito population, both vertical transmission and venereal transmission routes have been confirmed to maintain flavivirus infection [6, 7]. In vertebrate hosts, viruses are injected intradermally by mosquitoes during a new blood meal. Immune cells in the skin, such as dendritic cell subsets, monocytes, and macrophages, are permissive for initial flavivirus infection in hosts and subsequently disseminate to systemic tissues through blood circulation [8]. Enhanced mosquito vector transmission of flaviviruses can significantly contribute to their epidemic potential. For example, a single mutation within the NS1 protein has been shown to increase ZIKV infectivity and prevalence in *Aedes aegypti* and therefore could have facilitated ZIKV transmission during its epidemic in 2016 [9].

Despite significant progress in understanding various aspects of TMUV over the past decade, our knowledge of its mosquito transmission remains limited. Although TMUV has been identified in different mosquitoes, such as *Cx. pipiens*, and the vector competence of *Cx. pipiens* has been experimentally confirmed [10], the mechanisms of TMUV cycling within mosquito populations are still unknown. Additionally, it is unclear whether evolutionary variations have an impact on viral infectivity and prevalence in mosquitoes.

In this study, we established a *Cx. quinquefasciatus* model for TMUV and conducted a detailed analysis of the transmission routes of TMUV within mosquito populations, as well as the viral factors affecting TMUV infection in mosquitoes. These findings will contribute to a better understanding of the vector transmission of TMUV.

## Materials and methods

### Viruses

Both TMUV strains used in the present study were rescued from infectious clones [11, 12], and all virus stocks were prepared in BHK-21 cells. CQW1 (KM233707.1) is a duck-origin strain isolated in 2013, while the prototypical strain MM_1775 (JX477685.2) was isolated from *Cx. tritaeniorhynchus* in Malaysia in 1955.

The chimeric TMUV MM/CQ-3′UTR has been reported previously [13]. MM/CQ-E and MM/CQ-NS1 were generated by replacing the entire MM_1775-E gene or NS1 gene with the corresponding genes from CQW1 using reverse genetic techniques.

### Thoracic microinjection of TMUV in mosquitoes

At 7–10 days post-hatching, the mosquitoes were anaesthetized on a cold tray and fixed on their sides using forceps. Under a stereomicroscope (Phenix, China), the thorax of each mosquito was examined to identify a distinct “V”-shaped soft tissue area that lacks the protection of the mosquito cuticle. TMUV, diluted in DMEM to a specific titre, was microinjected into this thoracic region. The injection tip (RWD, China) was inserted to a shallow depth to avoid causing damage, with a total injection volume of 300 nL per mosquito. Following the injection, the mosquitoes were maintained under standard conditions of 28 ± 2 °C and 80% humidity. The survival of the injected mosquitoes was monitored and documented at 24–48 h post-infection. Seven days post-infection, live mosquitoes were collected and subjected to RT-qPCR analysis to assess the viral loads.

### Membrane blood feeding

Commercial defibrinated sheep blood or heat-inactivated duck, mouse, or rabbit blood (following a pretreatment procedure similar to a previously reported method [9]) was gently mixed with TMUV at a 1:1 ratio. The mixed blood solution was then added to the membrane feeding system for blood-sucking insects (Hemotek, USA), and the temperature was maintained at 37 ℃. Subsequently, the feeders were placed into mosquito cups containing female mosquitoes starved from water and sucrose for 1–2 days. After approximately 45 min of blood meal in the dark, the feeder was removed, and the engorged female mosquitoes were selected, transferred to new containers and kept under standard conditions. Eight days post infection, live mosquitoes were collected and subjected to RT-qPCR analysis.

For the persistent TMUV infection experiment, 180 female mosquitoes were selected and randomly divided into 6 groups, with 30 mosquitoes in each group. Blood with a virus titre of 10^5.5^ TCID_50_/mL was fed, and live mosquitoes were detected on days 7, 14, and 35. The experiment was limited to 35 days because mosquitoes began to die after this time point.

### Mosquito transmission of MM_1775 and CQW1

At 7–10 days post hatching, a total of 240 female mosquitoes were randomly divided into 6 groups, with 40 mosquitoes in each group. After starvation treatment for 1–2 days, the mosquitoes were orally infected with MM_1775 or CQW1 at a titre of 10^5.5^ TCID_50_/mL. At 4, 7, and 14 days post-infection, tissue samples from the mosquito midgut, head/legs, and salivary glands were collected. These samples were then subjected to RT-qPCR analysis to determine the virus infection rate (IR), dissemination rate (DR), and transmission rate (TR).

To determine the differences in mosquito vector competence between MM_1775 and CQW1, female mosquitoes were orally infected with MM_1775, TMUV MM/CQ-3′UTR, MM/CQ-E or MM/CQ-NS1 at a dose of 10^5^ TCID_50_/mL. At 8 days post-infection, the mosquitoes were killed to detect IR. To further confirm these results, 10^3^ TCID_50_/mL of each virus were microinjected into mosquitoes, and at 7 days post-infection, the mosquitoes were killed to detect IR.

### Venereal transmission

The experimental procedure for the venereal transmission study was similar to a previously reported method [6] with slight modifications. In brief, *Culex* mosquitoes were divided into 4 groups, consisting of two female groups and two male groups. After a 24-h starvation period, one group of virgin female mosquitoes and one group of male mosquitoes were injected with CQW1 at a concentration of 10^5^ TCID_50_/mL. The remaining two groups were injected with MM_1775. One day post-infection, the survival of the mosquitoes was recorded, and an equal number of naive female/male mosquitoes were assigned to each group to allow for mating. A piece of cotton mesh soaked in a 10% sucrose solution was provided for mosquito feeding (the sucrose solution was replaced daily) and placed on top of the cage to prevent oral contamination.

### Vertical transmission

For the assessment of vertical transmission, at 7–10 days post-eclosion, 40 female mosquitoes were microinjected with CQW1 virus at a dose of 10^5^ TCID_50_/mL. These infected females were then housed with male mosquitoes. To stimulate egg laying, anaesthetized Kunming mice were placed on top of the container for mosquito feeding and allowed to feed for approximately 30 min (the mosquitoes were starved for 24 h prior to engorgement). After laying eggs, a portion of the eggs was directly subjected to virus detection, while the remaining eggs were hatched, and the resulting larvae were raised to adulthood for subsequent virus detection.

### RNA extraction and RT–qPCR

Total RNA was isolated from the mosquitoes using a Total RNA Extraction Kit (Axygen, USA) per the manufacturer’s instructions. Subsequently, 1st-strand cDNA was transcribed using a HiScript III 1st Strand cDNA Synthesis Kit (Vazyme, Nanjing, China). To detect viral copies, RT–qPCR assays were performed using 2 × Taq SYBR Green qPCR Premix (Innovagene, Changsha, China) with a CFX Connect Real-Time PCR Detect System (Bio-Rad, USA) following the manufacturer’s protocols. The primers used for RT-qPCR are listed in Table 1.

**Table 1:** **RT-qPCR primers used to detect TMUV**

Primer	Sequence (5′–3′)
CQW1-E-qPCR-F	AATGGCTGTGGCTTGTTTGG
CQW1-E-qPCR-R	GGGCGTTATCACGAATCTA
MM_1775-NS5-qPCR-F	GAAATCGAATCTGCCAGGAC
MM_1775-NS5-qPCR-F	CCGCTCACCCAATACATC

### Quantification and statistical analysis

The RT-qPCR data are presented as the mean ± standard error of the mean (SEM). Using GraphPad Prism 9.5 software, statistical significance was assessed by Student’s* t* test, and significance was defined by a *P* value < 0.05 (*).

## Results

### *Cx. quinquefasciatus* model for TMUV infection through membrane blood feeding

**Figure 1:**
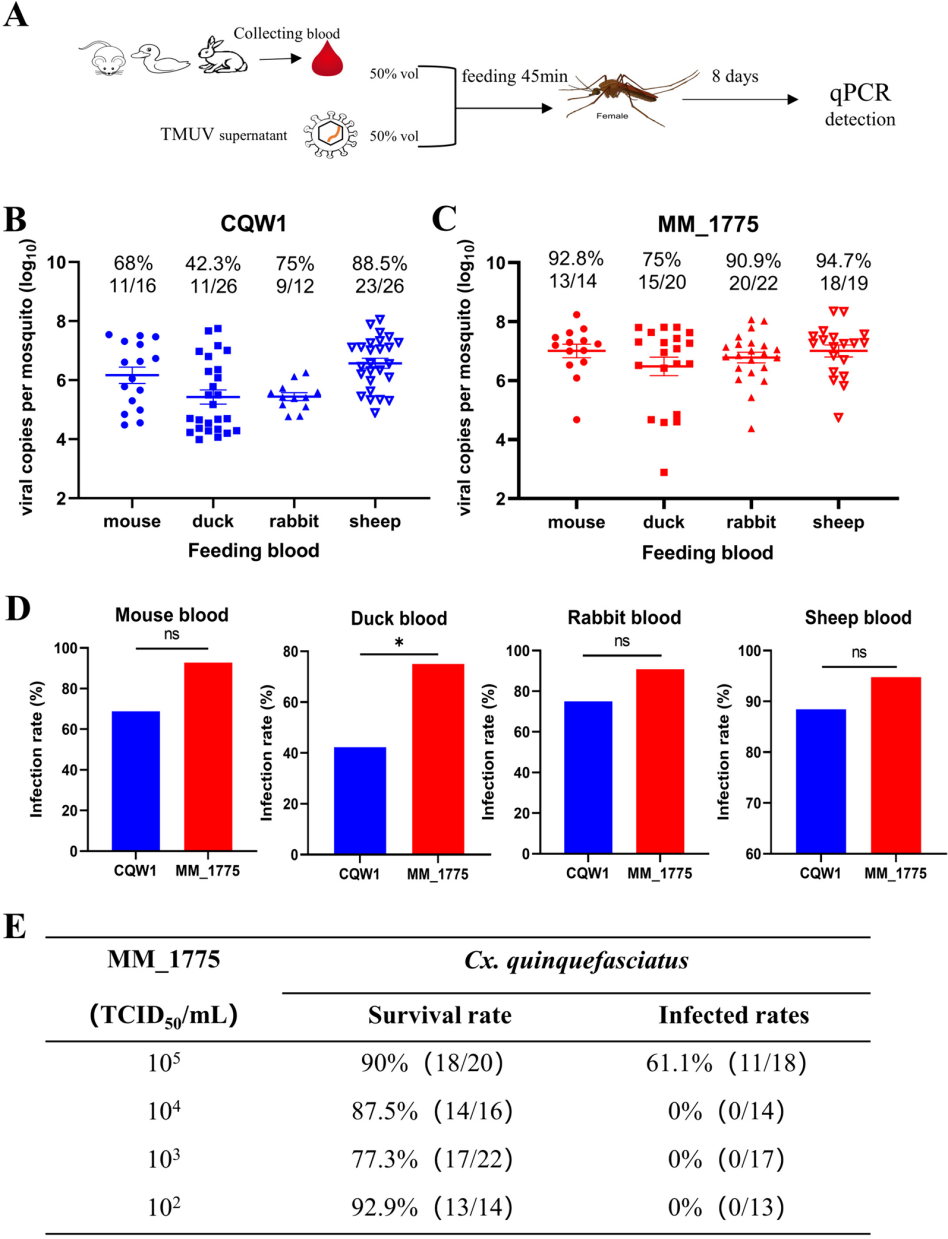
***Cx. quinquefasciatus*** **model for TMUV infection through membrane blood feeding.** **A** Female *Cx. quinquefasciatus* mosquitoes were orally infected with either the CQW1 or MM_1775 virus via different blood sources from mice, ducks, rabbits, or sheep. **B** At 8 days post-infection, the infection rates of CQW1 were determined using RT-qPCR. **C** The infection rates of MM_1775. **D** Infection rate comparison of MM_1775 and CQW1 from (**B**, **C**). **E** The minimum dose required for MM_1775 virus infection through membrane blood feeding was determined. Statistical significance: *P < 0.05.

To simulate the infection process through mosquito bites, we initially employed the membrane blood feeding method to establish a *Cx. quinquefasciatus* model for studying TMUV transmission. We tested four different sources of blood from various animal hosts (duck, mouse, rabbit, and sheep) for infection (Figure 1A). As shown in Figures 1B–D, when feeding on duck blood, the IR of the mosquito TMUV (MM_1775 strain) was significantly greater than that of the duck TMUV (CQW1 strain). This may be attributed to the interaction between the duck host and the viruses. MM_1775 also showed a slightly greater IR than did CQW1 when fed with blood from other sources, although the difference was not statistically significant. Nevertheless, the data indicate that all blood from different host sources is competent for TMUV infection through membrane blood feeding. When commercial fibre sheep blood was used, the *Culex* infection rates of CQW1 and MM_1775 were relatively high, and considering the convenience and easy access of blood, sheep blood was used for subsequent membrane blood feeding.

To determine the minimum dose of TMUV required to establish infection in *Culex* mosquitoes through membrane blood feeding, we performed a series of tenfold continuous dilutions of the MM_1775 virus solution (in DMEM) and mixed it with sheep blood for feeding. As shown in Figure 1E, most mosquitoes survived at 2 days post-infection. Surprisingly, an IR of 61.1% (11/18) was observed only at a dose of 10^5^ TCID_50_/mL, and no viral RNA was detected when the infection dose was less than 10^4^ TCID_50_/mL. These findings suggest that *Cx. quinquefasciatus* is competent for both the MM_1775 and CQW1 strains through membrane blood feeding.

### Compared with duck TMUV, mosquito TMUV exhibits greater infectivity in *Cx. quinquefasciatus*

**Figure 2:**
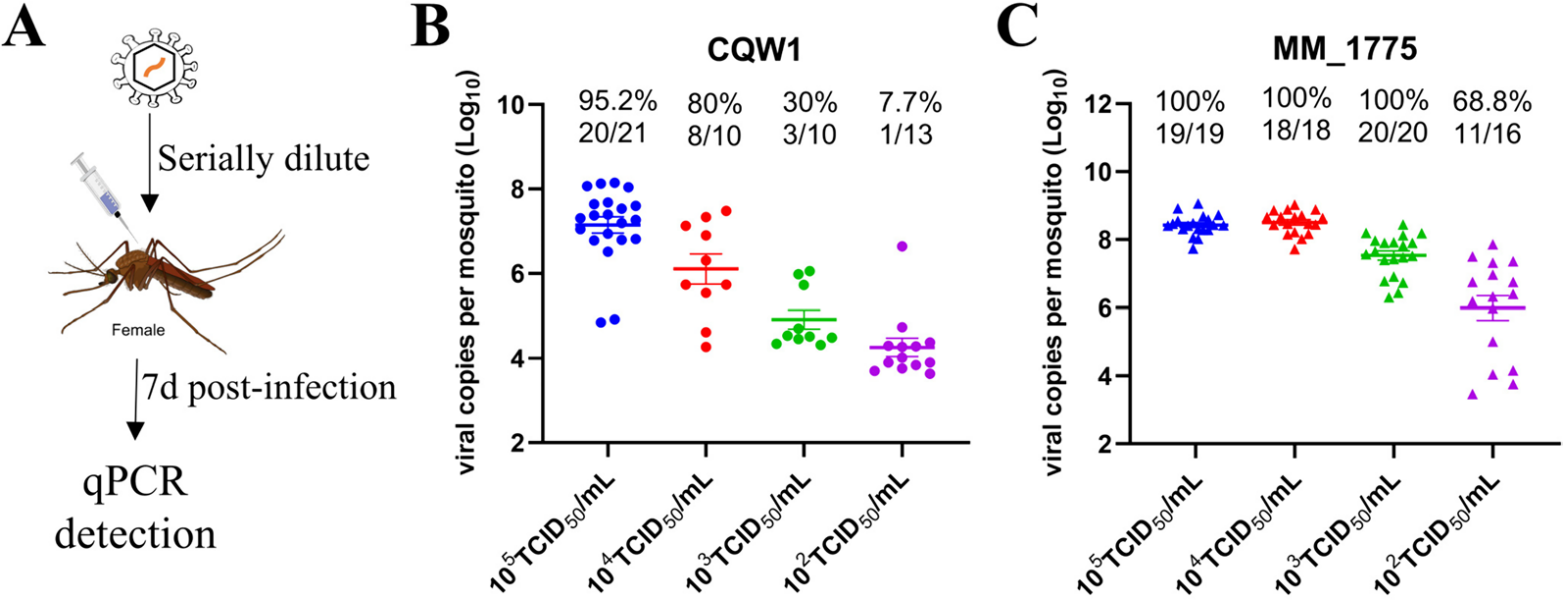
**Mosquito TMUV is more infectious than duck TMUV in Cx. quinquefasciatus.** **A** Female mosquitoes were infected with these two viruses through microinjection. At 7 days post infection, the infection rates were determined using RT-qPCR. **B** *Culex* mosquitoes were injected with CQW1 at various concentrations. **C** *Culex* mosquitoes were injected with MM_1775 at various concentrations.

Next, we used another artificial infection mode, microinjection, to determine the infective dose required for establishing infection (Figure 2A). As shown in Figure 2B, the IR of the CQW1 strain dramatically decreased from 95.2% (20/21) to 7.7% (1/13) when the infection dose decreased from 10^5^ TCID_50_/mL to 10^2^ TCID_50_/mL. In contrast, the MM_1775 strain exhibited greater potency, maintaining 100% IR when the infection dose was not less than 10^3^ TCID_50_/mL (Figure 2C). Even at a very low dose of 10^2^ TCID_50_/mL, 68.8% IR remained. These data suggest that MM_1775 is significantly more effective at infecting *Cx. quinquefasciatus* compared to CQW1 at the same dose.

Currently, there are limited data available on the vector transmission of TMUV. To better understand the effect of different virus strains on viral vector transmission, we compared the virus dissemination and transmission of the MM_1775 strain with those of the CQW1 strain. As shown in Table 2, viral dissemination of MM_1775 was consistently detected at all timepoints. In contrast, the dissemination of CQW1 viruses was detected only at later timepoints. Most importantly, only MM_1775 viruses were detected in the salivary glands of the mosquitoes, while no CQW1 virus RNA was detected under the same conditions. These results suggest that the mosquito-derived strain MM_1775 has a better transmission capability in mosquitoes than does the duck-derived strain CQW1.

**Table 2:** **Viral infection, dissemination, and transmission of CQW1 and MM_1775**

Days	IR		DR		TR	
	CQW1	MM_1775	CQW1	MM_1775	CQW1	MM_1775
4	100% (14/14)	90.9% (20/22)	0	15% (3/20)	0	0
7	100% (15/15)	73.7% (14/19)	33.3%(5/15)	14.3% (2/14)	0	50% (1/2)
14	76.9% (10/13)	100% (18/18)	20% (2/10)	27.8% (5/18)	0	20% (1/5)

IR (Infection rate): number of positive midgut samples/total number of female mosquitoes tested.

DR (Dissemination rate): number of positive head and leg samples/number of total samples.

TR (Transmission rate): number of positive salivary gland samples/number of total samples.

### Viral factors influencing the vector transmission of TMUV

**Figure 3:**
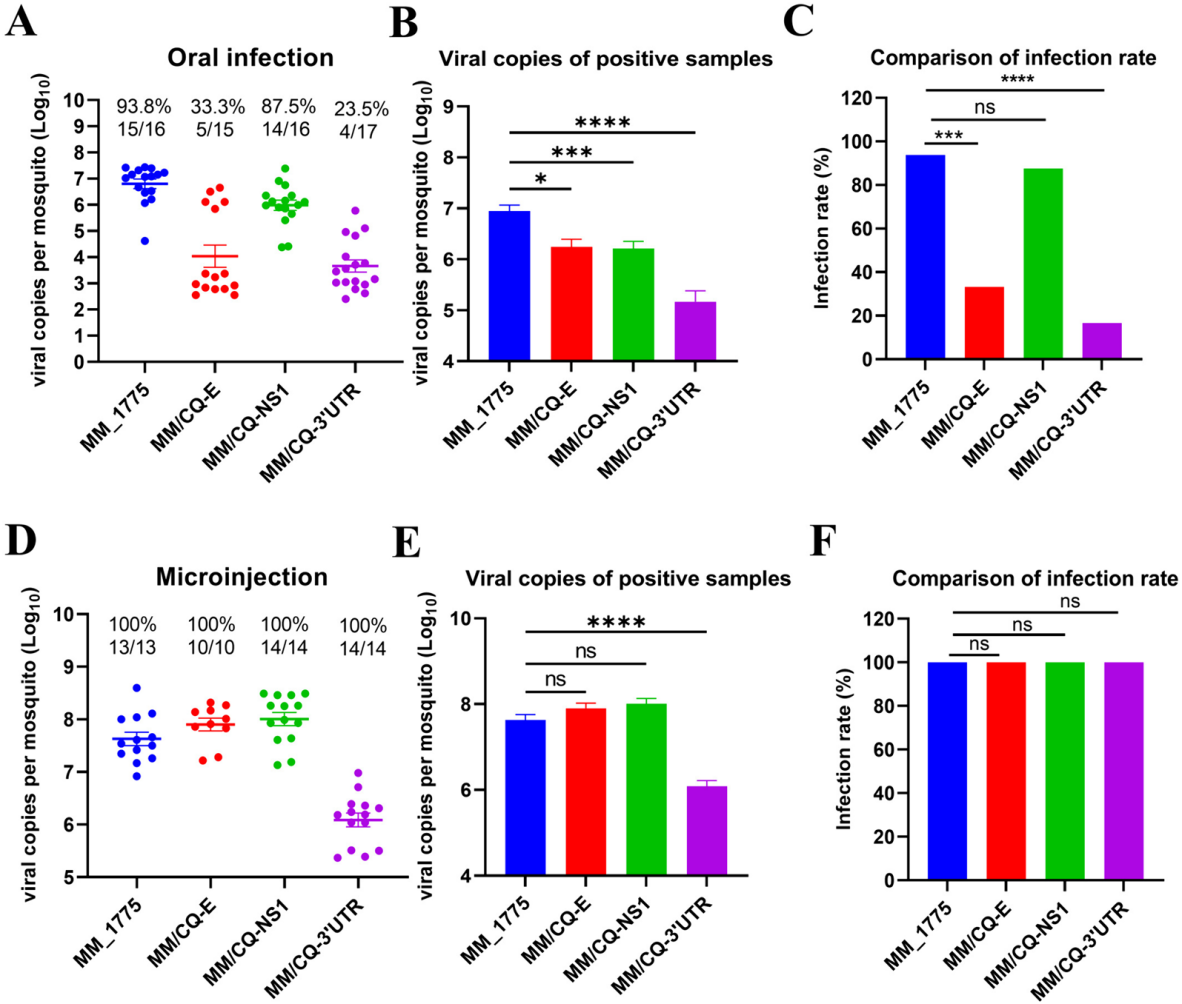
**Viral factors involved in vector transmission of TMUV.** **A** Infection rate of *Culex* mosquitoes orally infected with these chimeric viruses. **B** Viral copies of positive samples from (**A**). **C** Comparison of the infection rates from (**A**). **D** Infection rate of *Culex* mosquitoes infected with these chimeric viruses by microinjection. **E** Viral copies of positive samples from (**D**). **F** Comparison of the infection rates from (**D**). The data are presented as the means ± SEM. Statistical significance: *P < 0.05; **P < 0.01; ***P < 0.001; ****P < 0.0001.

To further investigate the viral determinants affecting TMUV transmission, a set of chimeric viruses was generated using MM_1775 as the backbone. Following feeding with 10^5^ TCID_50_/mL TMUV in a blood meal, the presence of virus was detected to calculate the IR at 8 days post-infection (Figures 3A–C). The IRs of the MM/CQ-E virus and MM/CQW1-3′UTR were 33.3% (5/15) and 23.5% (4/17), respectively, which were significantly lower than that of the wild-type MM_1775 (93.8%). However, the replacement of NS1 had little effect on IR in mosquitoes. When *Culex* mosquitoes were infected via microinjection, all chimeric TMUVs reached 100% IR, but the viral loads of the MM/CQW1-3′UTR strains were lower than those of the other strains (Figures 3D–F). The results suggest that variations in the E protein and 3′UTR, but not in NS1, are responsible for the differences in mosquito infection between the MM_1775 and CQW1 strains.

### Venereal transmission of TMUV in paired mosquitoes

**Figure 4:**
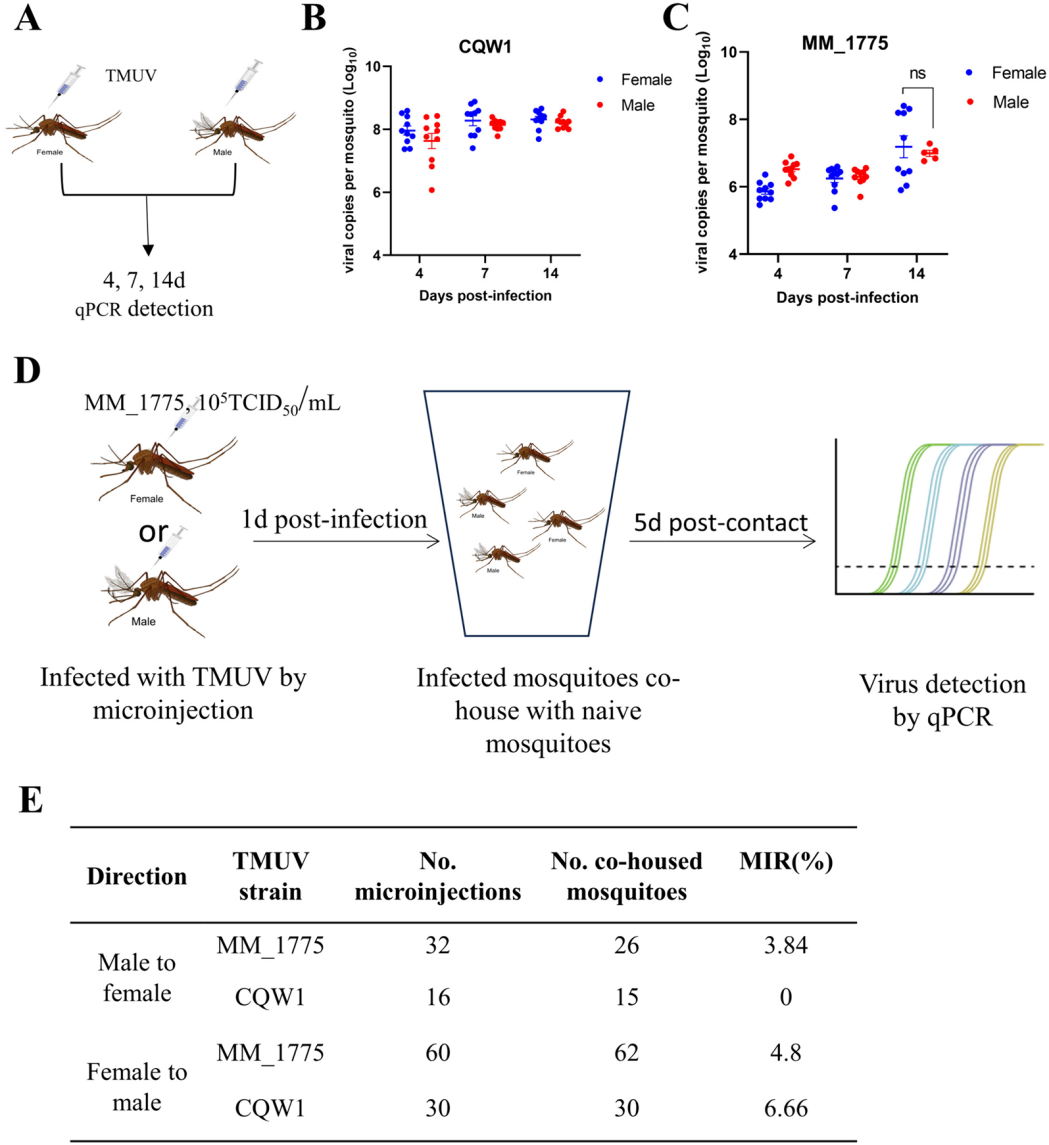
**Venereal transmission of TMUV in paired mosquitoes.** **A** To compare the sex of *Culex* mosquitoes during viral infection, female or male mosquitoes were infected with MM_1775 or CQW1 through microinjection. **B** The infection rate of female or male mosquitoes infected with CQW1. **C** The infection rate of female or male mosquitoes infected with MM_1775. **D** The experimental procedure for detecting venereal transmission. Female (or male) mosquitoes were infected with CQW1 or MM_1775 through microinjection. At 1 day post-infection, virgin males (or females) were introduced into the respective female (or male) infected cages to allow them to freely mate for 5 days. Finally, virus infection was determined using RT-qPCR. **E** The infection rate detected from (**D**). The data are presented as the means ± SEM.

In the natural cycle, mosquito-borne flaviviruses are transmitted by female mosquito bites. However, the possibility of male mosquitoes acting as virus reservoirs through sexual transmission cannot be excluded. Therefore, we measured the viral loads and IRs of TMUV in both female and male mosquitoes (Figure 4A). Similar levels of CQW1 viral loads were detected in both female and male mosquitoes on days 4, 7 and 14 post-infection (Figure 4B), with no significant difference in the IRs between the sexes of *Culex* mosquitoes. Similar results were observed for the mosquito TMUV MM_1775 strain (Figure 4C), indicating that the sex of *Culex* mosquitoes has no effect on TMUV transmission among mosquitoes.

To assess whether TMUV can be transmitted from female to male or from male to female mosquitoes, infected female or male mosquitoes were grouped into mating pairs with naive male or female mosquitoes, respectively (Figure 4D). Both the MM_1775 and CQW1 viruses exhibited female-to-male transmission, with minimum infection rates (MRRs) of 4.8% and 6.66%, respectively (Figure 4E). Male-to-female transmission was also detected at an MIR of 3.84% for the MM_1775 virus. These findings indicate that TMUV can be transmitted in both directions, from male to female as well as from female to male.

### Vertical transmission of TMUV in *Culex* mosquitoes

**Figure 5:**
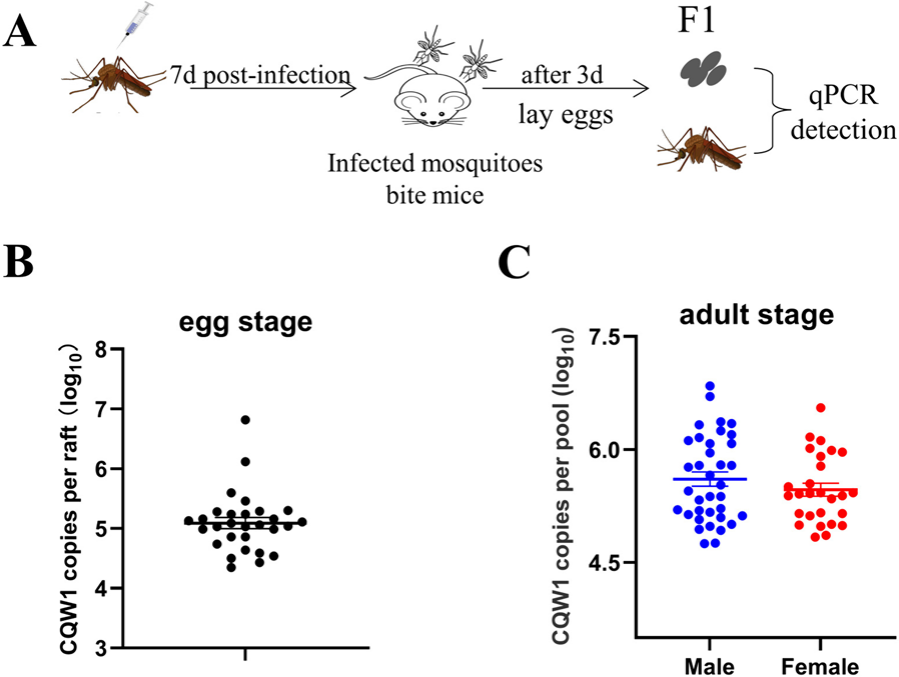
**Vertical transmission of TMUV in Culex mosquitoes.** **A** Female mosquitoes were microinjected with the CQW1 virus, and at 7 days post-infection, the mosquitoes were allowed to bite a blood meal. The engorged mosquitoes were continuously reared for oviposition. **B** A portion of mosquito eggs was collected to detect virus infection. **C** The remaining mosquito eggs were allowed to hatch, and the virus infection rate was detected at the adult stage. The data are presented as the means ± SEM.

It is believed that vertical transmission is the primary route for maintaining flaviviruses within mosquito populations. To verify whether TMUV can be vertically transmitted, female mosquitoes were infected with CQW1 viruses through microinjection. At 7 days post-infection, the infected mosquitoes were co-raised with mice to encourage mosquito bites and blood feeding, which would stimulate the females to lay eggs. A portion of the laid eggs was collected for virus detection, while the remaining eggs were used for hatching mosquito larvae (Figure 5A).

Among the collected mosquito egg samples, 13.8% (4/29) tested positive for TMUV, with a viral copy number of 10^5.091±0.9267^ (Figure 5B, Table 3). After hatching and reaching adulthood, 25.9% (7/27) of the female mosquito sample pools were positive for TMUV, with an MIR of 5.2% (7/135) and a copy number of 10^5.469±0.0873^ (Figure 5C, Table 3). Additionally, 47.2% (17/36) of the male mosquito sample pools were positive, with an MIR of 9.4% (17/180) and a copy number of 10^5.609±0.0953^. These findings indicate that viral RNA can be detected in both the egg stage and adult stage, suggesting that TMUV can be vertically transmitted within *Culex* mosquitoes.

**Table 3:** **Analysis of the infection rates of vertical transmission**

Strain	Stage	IR (%)	MIR (%)	RNA copies (log_10_)
CQW1	Egg (29/pool)	13.8 (4/29)	/	5.091 ± 0.9267
	Adult-Female (27/pool)	25.9 (7/27)	5.2 (7/135)	5.469 ± 0.0873
	Adult-male (36/pool)	47.2 (17/36)	9.4 (17/180)	5.609 ± 0.0953

IR: number of positive pools/number of total pools.

The minimum infection rate (MIR) was calculated as the number of positive pools/number of total mosquitoes.

### Persistent infection of TMUV in *Culex* mosquitoes

**Figure 6:**
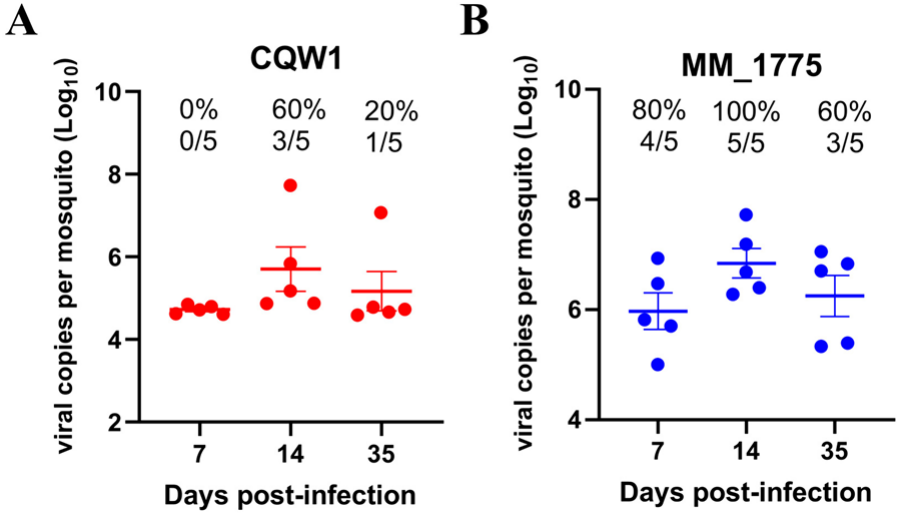
**Persistent infection of Culex mosquitoes with TMUV.** Female mosquitoes were microinjected with CQW1 or MM_1775 virus, and virus infections were detected at 7, 14, and 35 days post-infection. **A** CQW1 infection. **B** MM_1775 infection.

While it is well established that many arboviruses can cause persistent infections in mosquitoes, the details of the vector competency of *Cx. quinquefasciatus* for specific arboviruses are less well understood. Therefore, we determined the duration of TMUV infection in *Culex* mosquitoes. As expected, CQW1 displayed a lower IR than MM_1775, but viral RNA was still detected at 35 days post-infection (Figure 6A). For MM_1775, the IR increased from 80% (4/5) to 100% (5/5) between day 7 and day 14 after infection. Importantly, MM_1775 maintained 60% IR at 35 days post infection (Figure 6B). These results indicate persistent TMUV infection in *Culex* mosquitoes.

## Discussion

TMUV was first identified in Malaysia from *Cx. tritaeniorhynchus*. However, it was only occasionally reported in Southeast Asia in the following decades. In 2010, duck farms in most duck-breeding regions of China experienced an outbreak of an infectious disease known as duck egg-drop syndrome, and it was finally confirmed that TMUV was the cause. TMUV has since been detected in several species of *Culex* mosquitoes, including *Cx. tritaeniorhynchus*, *Cx. vishnui*, *Cx. quinquefasciatus*, *Cx. annulus*, and *Cx. pipiens* [14]. Therefore, *Culex* spp. have been proposed as the main transmission vectors for TMUV, particularly *Cx. tritaeniorhynchus* [15]. However, the vector competence of *Culex* mosquitoes has not been thoroughly characterized in the past decade.

In this study, we assessed the vector competence of *Cx. quinquefasciatus* and analysed the factors associated with vector transmission of TMUV in mosquitoes. Consistent with a previous report [15], only a high titre of TMUV (≥ 10^5^ TCID_50_/mL) resulted in successful infection when *Cx. quinquefasciatus* were challenged orally. This high titre requirement may be due to the lack of essential host factors or viral proteins that help mosquitoes acquire virions during the blood feeding process. This phenomenon has been widely reported for other mosquito-borne flaviviruses [8]. For example, ZIKV NS1 antigenemia promoted virion acquisition by mosquitoes, thereby facilitating transmission during the ZIKV epidemic in 2016 [9].

Compared to membrane blood feeding, microinjection is a more convenient and effective approach for infecting mosquitoes. However, the microinjection method cannot accurately reflect the process of infection during mosquito blood feeding because it skips the step of virus establishment in the midgut. However, it can be used as a supplement to membrane blood feeding methods and can be used to directly evaluate the level of virus replication in mosquitoes when membrane blood feeding data are insufficient. However, our data still indicate the successful establishment of a mosquito infection model for TMUV.

An increase in infectivity within mosquito vectors, leading to high epidemic potential, has been reported for several mosquito-borne viruses [9, 16, 17]. However, contrary to our expectations, the CQW1 strain showed significantly lower infectivity in mosquitoes than the MM_1775 strain. This result suggested that the outbreak of duck TMUV in China since 2010 may not be correlated with increased mosquito transmission of TMUV, and other routes of viral transmission (such as contact transmissibility) may play important roles in this process.

Flavivirus evolution shapes virus fitness in both vertebrate hosts and mosquitoes. Various viral determinants, such as C, E, NS1 and the 3′UTR, have been shown to influence flavivirus infectivity and transmission in mosquitoes [18]. In the present study, we revealed that both the E protein and the 3′UTR of TMUV contributed to differences in infectivity and transmission between MM_1775 and CQW1 in mosquitoes. The flavivirus E protein is a key envelope protein that controls virus attachment and entry, affecting viral host specificity and tissue/cell tropism. Additionally, the 3′UTR of flaviviruses, with its RNA structures and genomic variations, plays a role in host adaptation [19–21]. The 3′UTR is responsible for generating subgenomic flavivirus RNA (sfRNA), which can enhance mosquito transmission [22]. The structure of the 3′UTR determines the number and abundance of sfRNAs. In this study, we observed a significant decrease in the in vivo infectivity of TMUV in mosquitoes when the MM_1775 3′UTR was replaced with the CQW1 3′UTR, consistent with our previous findings [13] that the TMUV 3′UTR is responsible for cell-specific adaptation. However, further research is needed to elucidate the detailed mechanism underlying this phenomenon.

In conclusion, the present study successfully established the *Cx. quinquefasciatus* model for TMUV infection in the laboratory and investigated important factors associated with mosquito vector competence and transmission. These findings provide valuable insights for future research on TMUV mosquito transmission.

## Data Availability

All the data used to understand and assess the conclusions of this study are available in this published article. The raw data that support the findings of this study are available from the corresponding author upon reasonable request.
